# Correlation between systemic markers of inflammation and local synovitis in knee osteoarthritis

**DOI:** 10.22088/cjim.10.4.383

**Published:** 2019

**Authors:** Mansour Babaei, Yahya Javadian, Hossein Narimani, Mohammad Ranaei, Behezad Heidari, Hossein Basereh, Hemat Gholinia, Alireza Firouzjahi

**Affiliations:** 1 Mobility Impairment Research Center, Health Research Institute, Babol University of Medical Sciences, Babol, Iran; 2 Clinical Research Development Unit of Rouhani Hospital, Babol University of Medical Sciences, Babol, Iran; 3 Student Research Committee, Babol University of Medical Sciences, Babol, Iran

**Keywords:** Knee osteoarthritis, High sensitive C-reactive protein, Cytokines, Correlation, Synovitis, Systemic inflammation

## Abstract

**Background:**

In the elderly population joint swelling, effusion and pain indicate local synovitis and the presence of inflammation. At present, no serum marker has been shown linking to knee synovitis in KOA. With regard to serum high sensitive C-reactive protein (hsCRP) as a measure of inflammation, this study aimed to determine the association of systemic inflammation with local synovitis, as well as with pain and muscle strength in KOA.

**Methods:**

The study population was selected consecutively among patients with KOA presented to Ayatollah Rouhani Hospital Rheumatology Clinic with knee joint synovitis. The diagnosis of KOA was confirmed according to the American College of Rheumatology diagnostic criteria. Data regarding radiographic, demographic and biochemical characteristics were provided and IL-17, IL-10, TGF-ß and hsCRP in serum and synovial fluid (SF) were measured in all patients. Stepwse linear regression models were used to determine the correlation between SF- hsCRP as a measure of local inflammation with other systemic or local markers of inflammation.

**Results:**

A total of 40 patients (women 65%) with mean age 65.6+8.9 (49-86) years, mean BMI 27.7+3.7 (22-38) kg/m2, were analyzed. SF-hsCRP was positively correlated with serum hsCRP as well as serum and SF cytokines. Knee pain was positively associated with BMI and radiographic severity and negatively with quadriceps muscle strength (QMS) (r=-0.350, p=0.029). In stepwise linear regression analysis the SF-hsCRP was positively correlated with serum hs-CRP (r=0.769, p=0.001), SF-IL-17 (r=0.428, p=0.001) and negatvely with serum IL-10 (r=-0.316, p=0.002).

**Conclusion:**

These findings indicate that systemic markers of inflammation such as serum hsCRP, and IL-17 are associated with local inflamation in KOA.

Knee osteoarthritis (KOA) is a chronic slowly progressive disease and one of the most frequent cause of mobility impairment and disability in the elderly population (1). Localized synovitis in KOA is associated with pain, swelling and cartilage break down. Release of synovial molecules and damaged cartilage from synovial inflammation leads to systemic inflammatory response and liberation of proinflammatory cytokines into the systemic circulation (2). Clinically, sudden increase in knee joint pain particularly at night, and morning stiffness reflect synovitis (3). In arthroscopic studies, localized thickening of synovium can be detected in 50 % of the patients with KOA (4). High sensitive serum C-reactive protein (hsCRP) as a measure of systemic inflammation has been detected in synovial fluid (SF) of patients with KOA and was associated with inflammation of synovium and knee pain (5-7).

Interleukin -17 ( IL-17) which plays an important role in the coordination of immune cells at early stage of inflammatory process in rheumatoid arthritis, has been also detected in sublining layer of the joint synovium in osteoarthritis (8). This cytokine contributes to the development of cartilage inflammation through stimulation of chondrocytes and synovial fibroblasts (3, 8). In one study, 4 out of 152 patients with KOA (9.2%) had detctable IL-17 in SF suggesting a potential for inflammatory phenotype (9). In other studies of patients with osteoarthritis, the serum level of IL-17 was significantly higher than healthy controls and was positively correlated with WOMAC pain (10-12). The IL-17 is involved in the early phase of the inflammatory process in KOA and upregulates the production of metalloproteinases and decreases the level of proteoglycan (8, 10), whereas other cytokines such as IL-10 and TGF-ß supress inflammation and reduce the rate of osteoarthritis progression (12-15).

The diagnosis of KOA is based on clinical and radiographic features, and no appropriate laboratory marker has been identified to confirm the clinical diagnosis or display the presence of inflammatory process in osteoarthritis. Recognition of a serum marker to predict the presence of synovial inflammation in KOA would be helpful for both the diagnosis as well as for evaluation of treatment. Given hsCRP as a measure of inflammation, the aim of this study was to measure the level of hs-CRP in SF of patients with KOA and to determine its correlation with other systemic or local markers of inflammation.

## Methods

The participants of this study were selected consecutively among patients with KOA presented to Ayatollah Rouhani Hospital Rheumatology Clinic, because of knee joint pain and swelling with effusion. The diagnosis of KOA was confirmed according to the American College of Rheumatology diagnostic criteria based on clinical as well as radiological criteria (16). Individuals aged ≥ 40 years with knee pain for at least one month or longer were included. Exclusion criteria consisted of coexistent local or systemic inflammatory processes, history of inflammatory arthropathies, knee joint surgery. All patients underwent radiographic examination and the severity of KOA was determined according to the Kellgren and Lawrence grading scores (K-L) between 0 indicating normal radiograph to grade 4 indicating severe joint space narrowing and osteophyte in tibifemoral joint. The intensity of knee pain was assessed by both visual analogue scales (VAS), where 0 represented no pain and 100 mm indicated maximal knee pain, as well as by Western Ontario and McMaster University Osteoarthritis (WOMAC) pain scale consisted of 5 items. Each component of the WOMAC index was scored by Likert scale from 0-4 (17). The presence of knee joint synovitis was confirmed by clinical examination and joint aspiration by exclusion of other causes of joint effusios. Serum and synovial fluid in all patients were aspirated and the number of white blood cell count, polynuclear cells in the synovial fluid were determined immidiately. The levels of serum and SF -hsCRP were measured by ELISA method. The proinflammatory cytokines levels in SF and serum were determined by ELISA method using kits purchased from the Crystal Day company, China. Additional data were collected regarding biochemical, serological and demographic characteristics.

In statistical analysis, the association of SF-hsCRP as a measure of local inflammatory process was determined with serum hsCRP as well as with cytokines such as IL-17, IL 10, TGF-ß in the serum and SF. Stepwise linear regression models were used to determine correlation. Further analyses were applied to determine the association of pain with serum and SF-hsCRP, cytokines, radiographic severity, serum vitamin D and QMS. SSSP Version 22 were used for analysis. All patients gave informed consent and the proposal for this study was approved by the Ethics Committee of Babol University of Medical Sciences, Babol Iran.

## Results

A total of 40 patients (women, 65%) with mean age 65.6+8.9 (49-86) years, mean BMI 27.7+3.7 (22-38) kg/m2, were analyzed. Mean white blood cell count was 35+119 per ml SF. The levels of hs-CRP and cytokines in serum and SF are presented in table 1. As shown in table 1, the values of TGF-ß and IL-10 in SF were significantly higher than in serum, but the level of IL-17 in SF was nonsignificantly higher and the level of hsCRP was nonsignificantly lower in the SF as compared to serum. Radiographic evidence of grades 0-2 according to the K-L grading system was observed in 16 women and 11 men, and grades 3 and 4 were observed in 10 women and 3 men. The differences did not reach to a statistical level .Knee pain in joint with synovitis was significantly higher than contralateral joint without synovitis (47.4+3.86 vs 13.2+2.89, p=0.001). The mean serum 25-hydroxyvitamin D was 37+18.5 ng/ ml, the ESR was 25+16 mm/h, the serum calcium was 9+0.38 mg/dl, the phosphate was 3.5+0.2 mg/dl the serum, uric acid was 4.28+1.1 mg/dl. The mean serum hsCRP concentration in SF and serum was 2.31+1.59 and 2.60+2.03 mg/ml, respectively (p=0.34). The mean IL- 17 concentration in SF was 313+70 (105-1182) pg/ml. The serum levels of proinflammatory cytokines in SF and serun are shown in table 1.

**Table 1:** Serum and synovisl fluid levels of high sensitive serum C-reactive protein (hsCRP), and proinflammatory cytokines in patients with knee osteoarthritis and synovitis

**Variables **	**Synovial fluid** **Mean** **+** **SD (range)**	**Serum** **Mean** **+** **SD**	**p value**
Hs-CRP, mg/l	2.52+1.59 (0.92-8.95)	2.91+2.04 (1.09-10.1)	0.34
IL-17, pg/ml	313+70 (105-1182)	279+211 (105-1182)	0.33
TGF-β, ng/ml	2961.7+481 (416-3230	822+503 (256-2601)	0.001
IL-10, pg/ml	427+153 (129-697)	280.1+145 (166-800)	0.001

The results of correlative analysis between SF-hs-CRP and cytokines by linear regression analysis are shown in table 2. In stepwise linear regression analysis, the SF-hsCRP was positively correlated with serum hs-CRP (p=0.001) and SF-IL-17 (p=0.001) but negatively correlated with serum IL-10 (p=0.002) (table 3). There was no correlation between knee pain and serum and synovial hs-CRP or cytokines. However, knee pain was positively associated with severity of radiographic abnormalities and BMI.

**Table 2:** Correlation* between high sensitive C-reactive protein in synovial fluid (SF-hsCRP) and serum hsCRP as well as synovial and serum cytokines in patients with knee osteoarthritis and local synovitis

	**Serum**				**Synovia** **l fluid**		
	**hsCRP**	**IL-17**	**TGF-b**	**IL-10**	**IL-17**	**TGF-b**	**IL-10**
SF hsCRP	0.795	0.464	0.394	-0.357	0.685	0.462	-0.474
pvalues	0.001	0.003	0.012	0.024	0.001	0.003	0.024

* Pearson correlation test was used

**Table 3:** Stepwise linear regression analysis in relation to the association between synovial fluid (SF) high sensitive C-reactive protein (CRP), cytokines and body mass index (BMI)

**Variables**	**Unstandardized Coefficients**		**Standardized Coefficients**	**P value**
	**B**	**Std. Error**	**Beta**	
Serum hsCRP	0.606	0.083	0.769	0.000
SF-IL17	0.010	0.002	0.428	0.000
Serum-IL10	-0.003	0.001	-0.316	0.002

## Discussion

These findings indicate that in patients with KOA, SF-hs-CRP correlates positively with serum hs-CRP and SF-IL-17, and negatively with serum IL-10. A positive correlation between serum and SF- hs-CRP suggests a potential for serum hs-CRP in predicting local inflammatory process in KOA. Thus, in patients with localized synovitis, changes in severity of synovitis may be predicted by changes in serum hs-CRP concentration. 

The results of this study are consistent with the results of previous studies (5, 18, 19), and indicate that serum hs-CRP can be considered as a measure for detection of inflammatory state in KOA. Elevated level of SF- IL-17 and its positive correlation with inflammatory process suggests a pathophysiological role for this cytokine in the development of inflammatory process. 

In patients with rheumatoid arthritis, IL-17 cooridinates local inflammation and induces proinflammatory cytokines to perpetuate an inflammatory reaction, and contribute to the development of cartilage and synovial inflammation and bone destruction (8). Thus, production of IL-17 from synovial membrane of osteoarthritis joint may indicate a role for this cytokine in the initiation of synovitis in KOA (8).

In a study by Snelling et al. high level of SF-hsCRP in KOA was suggestive of synovial inflammation as well as the presence of an ongoing inflammatory process (9).Chen et al. have shown an association between SF-IL-17 and radiographic severity in patients with KOA (20). In this study, the level of IL-10 in SF was significantly higher than in serum. This may be contributed to the release of synovial molecules and damaged cartilage from the inflammed synovium in response to systemic inflammation (13). IL-10 is an anti-inflammatory cytokine which regulates hemostasis of the immune system and slows progression of KOA (13). The negative correlation between SF-hs-CRP and serum IL-10 indicate that increasing serum IL-10 is associsted with decreased SF-hs-CRP, which is consistent with the anti-inflammatory and protective characteristics of IL-10 (21). A similar relationship has been reported in rheumatoid arthritis. Treatment of rheumatoid arthritis with methotrexate or gold salts is associated with increased level of serum IL-10 and decreased level of serum CRP (22, 23)

In the present study, lack of correlation between pain and hs-CRP or cytokines may indicate contribution of factors other than synovitis to the development of pain in KOA (24, 25). Limitations of this study include, absence of a control group for comparison of serum hsCRP or cytokines between KOA and healthy controls. The diagnosis of synovitis was made by clinical examination without histlogic examination. However, pain, swelling and aspiration of synovial fluid are highly suggestive of synovitis. The study design was cross-sectional and so the observed association does not indicate causality. Detection of a significant correlation between serum and SF- hsCRP in this study should be considered as a strength, because data in this context are scarce. Nonetheless, lack of a control group prohibited to specify a cutoff level for serum hsCRP to discriminate patients with and without synovitis. 

In conclusion, the findings of this study indicate an association between systemic inflammatory process and local synovitus in patients with KOA. Thus, detection of an association between KOA and systemic inflammation would be possible by serum hs-CRP measurement. However,this issue requires further investigations. A longitudinal follow-up study with measurements of serum and synovial hs-CRP at regular intervals, and detection of a significant correlation between changes in serum and SF hs-CRP concentrations might provide further documents for this purpose.
